# HIP Fracture Evaluation and Management

**DOI:** 10.1155/2019/2717518

**Published:** 2019-11-27

**Authors:** Sumon Nandi, Paul Dougherty, Gary Gruen, Nabil Ebraheim

**Affiliations:** ^1^The University of Toledo College of Medicine and Life Sciences, Toledo, OH, USA; ^2^The University of Florida College of Medicine—Jacksonville, Jacksonville, FL, USA; ^3^University of Pittsburgh, Pittsburgh, PA, USA

Hip fracture incidence is over 250,000 per year in the United States and is expected to grow to 850,000 by the year 2040 [1]. The severity of a hip fracture is underscored by the mortality rate of 20 to 30% in the year following injury [1, 2]. Most hip fractures occur in those older than 50, a demographic in which there is a higher prevalence of comorbidities [3]. As a result, minimizing perioperative adverse events while maximizing postoperative function and reoperation-free survival is essential in hip fracture patients. These aims not only seek to optimize quality of care in a large patient population, but also control cost as hip fractures comprise the majority of orthopaedic trauma-associated health care expenditures [4].

In this issue, investigators discuss preoperative optimization, intraoperative surgical technique, discharge planning, and management of postoperative complications in hip fracture patients. By offering guidance to surgeons throughout the entire episode of care, from hospital admission to follow-up, we hope to provide a broad-based overview of hip fracture evaluation and management.

T. S. Moores and colleagues describe an effective protocol for reversing the effect of warfarin in hip fracture patients that decreases time to the operating room (OR) following presentation. Morbidity and mortality following hip fracture are mitigated by decreasing time to OR following injury, so Moores' findings are impactful.

Subtrochanteric femur fractures pose a clinical challenge, not only due to the technical difficulty of reduction and fixation, but also the high rate of nonunion. C. Jackson et al.'s text offers technical pearls within the framework of an anatomic and biomechanical discussion that is helpful to surgeons as they strive to minimize these issues.

S. R. Konda and his coauthors present use of a validated risk assessment tool to predict discharge disposition and readmission among hip fracture patients. Strategies for optimization of discharge planning are offered to minimize costs associated with the hip fracture episode of care.

Nonunion following hip fracture fixation in the elderly is highly consequential as revision surgery may be required in this medically frail and functionally compromised patient population. J. F. Kellam et al. provide a discussion of optimal methods for hip fracture fixation to prevent nonunion, a rubric for determining the cause of nonunion, and revision surgery techniques to treat nonunion.

Nearly all orthopaedic surgeons care for hip fracture patients. In the issue to follow, we aim to present papers that are immediately applicable to the clinical practices of our broad audience.
